# New insights into mammalian sex chromosome structure and evolution using high-quality sequences from bovine X and Y chromosomes

**DOI:** 10.1186/s12864-019-6364-z

**Published:** 2019-12-19

**Authors:** Ruijie Liu, Wai Yee Low, Rick Tearle, Sergey Koren, Jay Ghurye, Arang Rhie, Adam M. Phillippy, Benjamin D. Rosen, Derek M. Bickhart, Timothy P. L. Smith, Stefan Hiendleder, John L. Williams

**Affiliations:** 1 The Davies Research Centre, School of Animal and Veterinary Sciences, University of Adelaide, Roseworthy, South Australia Australia; 2 Genome Informatics Section, Computational and Statistical Genomics Branch, National Human Genome Research Institute, Bethesda, MD USA; 3 Center for Bioinformatics and Computational Biology, Lab 3104A, Biomolecular Science Building, University of Maryland, College Park, MD USA; 4 Animal Genomics and Improvement Laboratory, ARS USDA, Beltsville, MD USA; 5 Cell Wall Biology and Utilization Laboratory, ARS USDA, Madison, WI USA; 6 US Meat Animal Research Center, ARS USDA, Clay Centre, NE USA

**Keywords:** Genomes, Livestock, Bovine, Sex chromosomes, Pseudoautosomal region

## Abstract

**Background:**

Mammalian X chromosomes are mainly euchromatic with a similar size and structure among species whereas Y chromosomes are smaller, have undergone substantial evolutionary changes and accumulated male specific genes and genes involved in sex determination. The pseudoautosomal region (PAR) is conserved on the X and Y and pair during meiosis. The structure, evolution and function of mammalian sex chromosomes, particularly the Y chromsome, is still poorly understood because few species have high quality sex chromosome assemblies.

**Results:**

Here we report the first bovine sex chromosome assemblies that include the complete PAR spanning 6.84 Mb and three Y chromosome X-degenerate (X-d) regions. The PAR comprises 31 genes, including genes that are missing from the X chromosome in current cattle, sheep and goat reference genomes. Twenty-nine PAR genes are single-copy genes and two are multi-copy gene families, OBP, which has 3 copies and BDA20, which has 4 copies. The Y chromosome X-d1, 2a and 2b regions contain 11, 2 and 2 gametologs, respectively.

**Conclusions:**

The ruminant PAR comprises 31 genes and is similar to the PAR of pig and dog but extends further than those of human and horse. Differences in the pseudoautosomal boundaries are consistent with evolutionary divergence times. A bovidae-specific expansion of members of the lipocalin gene family in the PAR reported here, may affect immune-modulation and anti-inflammatory responses in ruminants. Comparison of the X-d regions of Y chromosomes across species revealed that five of the X-Y gametologs, which are known to be global regulators of gene activity and candidate sexual dimorphism genes, are conserved.

## Background

The sex chromosomes evolved from ancestral autosomes in dioecious lineages and have become extensively differentiated in structure and gene content [1, 2]. Mammalian X chromosomes are mainly euchromatic with a similar size and structure among species, and have retained most of the ancestral X genes [3, 4]. In contrast, Y chromosomes have undergone substantial evolutionary changes, accumulated male specific genes and genes involved in sex determination, and have lost 95% of the ancestral genes [5]. As a consequence, the Y chromosome is much smaller than the X chromosome and comprises mainly the pseudoautosomal (PAR), X-degenerate (X-d) and ampliconic regions [6].

The PAR is conserved on the X and Y, pairing and recombining at meiosis [7]. Most mammals have a single PAR region but the human sex chromosomes are an exception with a second PAR at the distal ends of the X and Y chromosomes [8]. The PAR plays an essential role in normal sexual development and loss of the PAR is associated with male sterility in humans [9]. Despite its critical role in fertility and disease, the PAR is one of the least well-characterised parts of most mammalian genomes. Previous studies have described the genes at the bovine pseudoautosomal boundary and PAR gene content [10–12], but currently there is neither a complete gene map nor a precise size available for the bovine PAR.

The X-d regions of the Y chromosome contain single-copy genes, pseudogenes, which appear to be surviving relics of the autosomes from which the Y chromosome evolved, and ampliconic regions, which consist of large heterochromatic blocks rich in repetitive sequences [9, 13, 14]. Both the X-d and ampliconic regions are male-specific. Unlike the highly conserved PAR, the structure and gene content of the X-d regions differ among mammalian species. The human X-d regions are interrupted by several large blocks of ampliconic sequences, while the X-d regions of chimpanzee include a single ampliconic block [9, 15]. A study of bovine Y chromosome gene expression has contributed information on genes in X-d regions [16], but interpretation of the data is limited by the relatively poor quality of the available Y chromosome assembly.

The complex and highly repetitive Y ampliconic regions are difficult to assemble, particularly from short sequence reads. Many mammalian genome sequencing projects have used a female subject to avoid having to resolve X and Y haplotypes, and therefore do not include the Y chromosome. Only a few species, including human [6], chimpanzee [15], rhesus macaque [17], mouse [18], pig [19] and horse [20], have well characterised and assembled Y chromosomes.

The *Bos taurus taurus* reference genome assembly Btau_5.0.1 [21] (NCBI Project ID:20275) was assembled from short and long sequence reads of BAC clones and contains a Y chromosome sequence. A *Bos taurus indicus* Y chromosome was created by alignment of short read sequences to Btau_4.0 [22], and therefore will be missing any larger indicine-specific features. These sex chromosome assemblies are incomplete and inconsistent, hindering studies on sex chromosome evolution and the dissection of the molecular architecture of sexually dimorphic phenotypic traits.

In the present article, we report high quality assemblies of bovine X and Y chromosomes, created from long read sequences and optical mapping data, using a trio binning approach [23] that exploited the high level of DNA sequence divergence between the two subspecies of domestic cattle [24–26], *Bos taurus taurus* and *Bos taurus indicus*. We present a detailed gene map of the complete bovine PAR and X-d regions from these assemblies and discuss the evolutionary changes and functional aspects in these regions in comparison with other mammals.

## Results

### Assembly and annotation of the cattle sex chromosomes

The bovine X and Y chromosomes were assembled from whole genome sequence of a hybrid male with a *Bos taurus taurus* (Angus) sire and a *Bos taurus indicus* (Brahman) dam [23] (see URLs). The assembled chromosomes presented here are the Brahman X chromosome which comprises 146 Mb in 106 contigs with 983 genes, and the Angus Y chromosome which comprises 16 Mb in 67 contigs with 51 unique genes (Table 1 and Additional file 1: Table S1). These sequence assemblies have been deposited at NCBI (X: CM0011833.1; Y: CM0011803.1). The full length of the cattle Y chromosome has been estimated as ~ 50 Mb, at least half of which is in the highly repetitive region [27]. As in other species [19, 20, 28], even with long read sequencing, we could not assemble the ampliconic highly repetitive region [27] or the heterochromatic regions. Full annotation of the Brahman X and Angus Y chromosomes are available from Ensembl release v97 (UOA_Brhaman_1 and UOA_Angus_1). Analysis of the PAR and X-degenerate regions are presented below.

**Table 1:** Length and number of gaps for Mammalian Sex chromosomes

	X Length (bp)	X Gaps	Y Length (bp)	Y Gaps
Cattle-Brahman/Angus	146,049,346	91	15,624,455	69
Cattle-Nelore	82,205,613	10,873	14,991,264	35,040
Cattle-Hereford	139,002,886	55	38,719,986	18
Water buffalo	143,477,029	65	–	–
Goat	115,943,529	319	–	–
Sheep	132,936,813	2968	–	–
Pig	125,778,992	10	15,567,420	12
Dog	123,180,702	1032	–	–
Horse	127,806,490	300	8,967,074	560
Human	154,893,106	28	26,415,094	55

### Identification of the cattle PAR

**Fig. 1:**
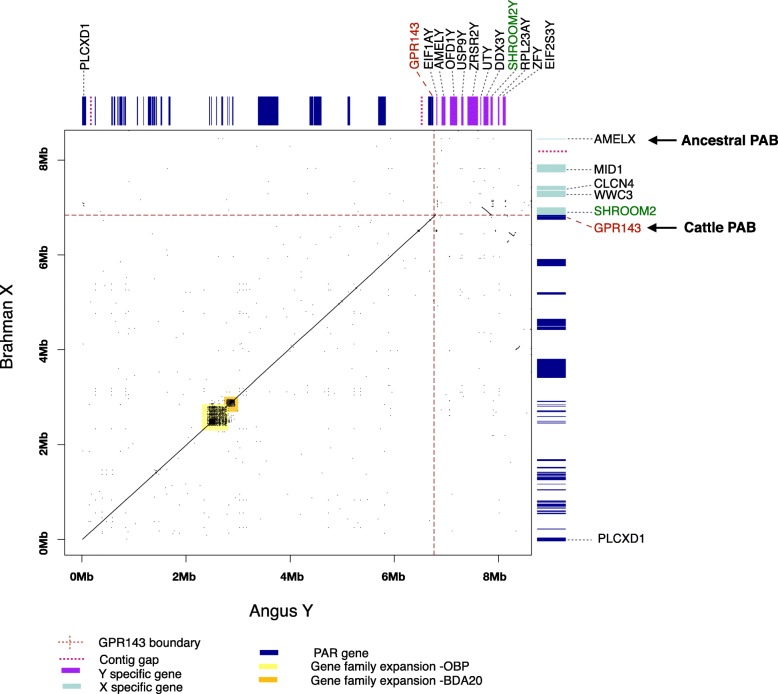
Comparison of the Brahman X and Angus Y cattle chromosome pseudoautosomal regions (PARs) and adjacent sequence. The x-axis is Angus Y chromosome and y-axis is Brahman X chromosome. The cattle PAR is located between *PLCXD1* and *GPR143*, 3286 bp distal to *GPR143*. The X and Y chromosome PAR genes (dark blue) share the same gene content and gene order. Gene families *OBP* and *BDA20* are highlighted in yellow and orange. The X chromosome PAR is assembled in one contig while the Y chromosome PAR has two contig gaps. *SHROOM2* is located distal to the pseudoautosomal boundary (PAB) on the X chromosome, which is found inverted on the Y chromosome X-d1 region

Alignment of assembled Brahman X and Angus Y chromosomes to each other identified a 6.8 Mb region with 99% sequence identity that extends from the start of the assembled X chromosome sequence (CM0011833.1) to 2933 bp distal to *GPR143*, after which sequence identity decreases to 86% for 348 bp and then drops abruptly to ~ 15% for the next 1 Mb (Fig. 1). The X chromosome PAR is assembled in one contig while the Y chromosome PAR only has two contig gaps. This enabled us to precisely define the PAR boundary and size. The PAR on the Brahman X and Angus Y chromosomes contained 31 genes in the same order. Of these, 29 are single-copy genes and two are multi-copy gene families, *OBP*, which has 3 copies and *BDA20,* which has 4 copies (Additional file 1: Table S4). The Brahman X chromosome PAR contains 12 genes that are missing from the proximal end of the X chromosome in the current Hereford reference genome ARS-UCD1.2 (Additional file 1: Table S3).

### Identification of cattle X-degenerate regions

**Fig. 2:**
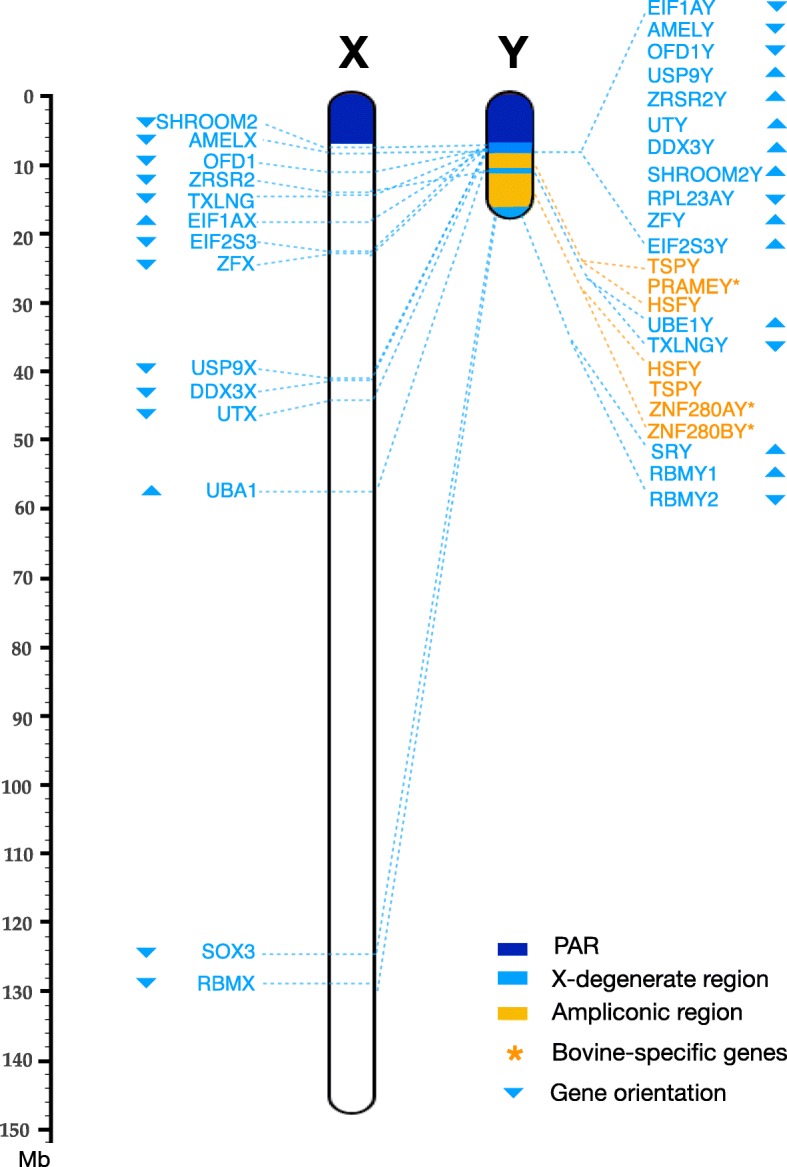
A gene map of X-Y homologous genes outside the cattle PAR. X-degenerate (X-d1, Xd2a and X-d2b) and ampliconic regions are indicated. 14 X-Y paired genes and 6 multi-copy genes are shown. Of 11 genes located in X-d1 (light blue), all but *RPL23AY* have homologous on the chromosome 19. Gene orientations are not consistent for X-Y paired genes. Multi-copy genes (yellow) and bovine-specific Y genes (asterisk) are indicated

Additional genes outside the PAR showed between 60 and 96% sequence identity between the X and Y chromosomes and are located in X-degenerate regions of the Y chromosome. The first of these regions, X-d1, is located distal to the PAR and spans 1.48 Mb, between 6.84 Mb and 8.32 Mb. X-d1 contains 11 single-copy protein coding genes. The corresponding region on the X chromosome spans 35 Mb. and contains 10 X-d1 homologues in a different order but misses RPL23AY, which is located on chromosome 19 (Fig. 2). A 3 Mb ampliconic region immediately distal to X-d1 contains the male-specific Y (MSY) gene families *PRAMEY*, *TSPY*, and *HSFY.* At the distal end of the ampliconic region, the second X-degenerate region, X-d2a, spans 1.63 Mb and contains two single copy genes, *UBE1Y* and *TXLNGY*. The X chromosome homologs of these two genes are separated by a 44 Mb interval that contains 285 X chromosome-specific genes. Distal to X-d2a lies a 4.5 Mb ampliconic segment containing the bovine specific MSY genes ZNF280AY and ZNF280BY, which are equivalent to TSPY and HSFY found in other species. The copy numbers of multi-copy MSY gene families are listed in Additional file 1: Table S2 and the complex arrangement of multi-copy genes is presented in Additional file 1: Figure S3. The distal end of chromosome Y contains the third X-degenerate region, X-d2b, which extends over 1.3 Mb and includes SRY and two copies of RBMY. The X chromosome homologs of these, *SOX3* and *RBMX*, are located in a 5 Mb segment at the distal end of the X chromosome.

### Comparison of sex chromosome structure in mammals

Alignment of the Brahman X chromosome with the current *Bos taurus taurus* (Hereford) cattle reference sequence (ARS-UCD1.2) revealed a 4 Mb inversion as a major structural difference. In both assemblies this inverted region ends at contig breakpoints (Additional file 1: Figure S1a). Alignment of the Brahman X chromosome with the water buffalo X chromosome [29] revealed a high level of co-linearity, with one large inversion and five small inversions at the distal end of the chromosome (Additional file 1: Figure S1b). The Brahman and water buffalo X chromosomes are 30 and 25 Mb longer, respectively, than the goat X chromosome, which consists of two scaffolds with a combined length of 116 Mb [30]. The goat X chromosome shows excellent co-linearity overall with the sheep X chromosome (Additional file 1: Figure S1c-d) but both showed numerous break points and several inversions, particularly on the short arm, in comparison with the Brahman and water buffalo X chromosomes. Non-ruminant mammalian X chromosomes, i.e. human, pig, dog and horse, revealed a striking similarity in the pattern of rearrangements in comparison to the *Bos taurus indicus* (Brahman) X chromosome (Additional file 1: Figure S1e-h). These consisted predominantly of 5 large inversions.

Alignment of the Angus Y chromosome assembly with pig, horse and human Y chromosomes showed limited co-linearity which was confined to the PAR and X-degenerate regions (Additional file 1: Figure S2a-c).

### Gene content and order of the mammalian PAR

**Fig. 3:**
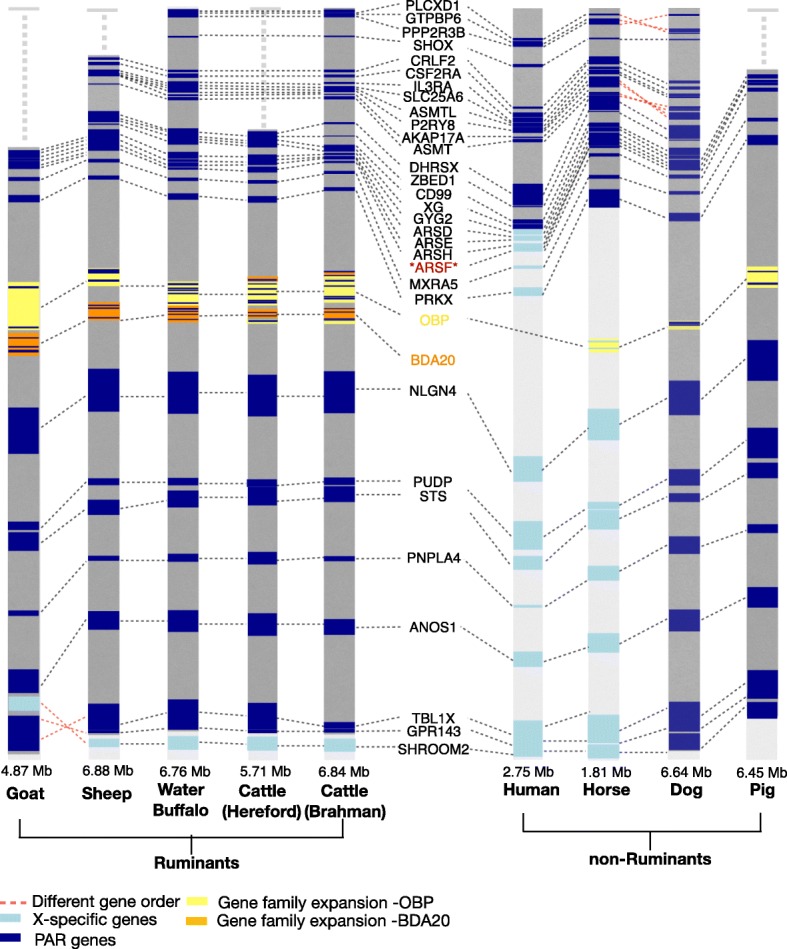
Comparison of the X chromosome PAR genes in cattle and other mammals. PAR genes (dark blue) and X specific genes (light blue) are indicated for each species. Red dashed lines represent genes with different order relative to the Brahman cattle. The pseudoautosomal boundary gene is the same in all ruminants except goat which has an inversion. Copy number of *OBP* and *BDA20* varies among species. The copy number of OBP and BDA20 are listed in Additional file 1: Table S4. *BDA20* is ruminant specific whereas *OBP* is found in all mammals except human. Assemblies for pig, Hereford cattle, sheep and goat show a truncated PAR. The numbers above each species name represent the approximate PAR size

There is a very high level of conservation of synteny among mammalian PARs (Fig. 3). PLCXD1 is the most proximal PAR gene in human, horse, Brahman cattle and water buffalo. At their proximal ends, the PAR regions in the Hereford cattle reference genome, and sheep, goat and pig assemblies are truncated distal to *DHRSX*, *CLRF2*, *CD99* and *GYG2* respectively (Additional file 1: Table S3). At their distal end, the pig and dog PAR extend beyond *GPR143* with a boundary distal to SHROOM2. In comparison to all the other species, the goat PAR has an inversion of three genes (*TBL1X*, *GPR143*, *SHROOM2*) close to the ruminant PAR boundary. This region is contained in one contig of the goat assembly and may thus be a contig orientation error, rather than a goat-specific rearrangement. The human sex chromosomes are an exception amongst mammals and have PARs at the proximal and distal ends [8]. The PAR1 in human is equivalent to the single PAR of other mammalian species, but is much shorter, with a distal boundary proximal to *XG*. The PAR of horse is the shortest with the distal boundary at *PRKX* (Fig. 3).

### PAR gene family expansions in different lineages

The *OBP* gene family, which is distal to *PRKX* in all species, is within the PAR of all ruminants, pig and dog, but is outside the PAR of horse, and is missing from the human X chromosome. This gene family is expanded in ruminants (Fig. 3). The *BDA20* gene family is immediately distal to the OBP family and present in all ruminants for which data are available, including Yak [31], Deer [32] and Chiru [33], but is not found in other mammals (Fig. 3). The *BDA20* family shows differential expansion in the different ruminant species, with two or more copies with 74–91% nucleotide sequence identity at mRNA level in cattle [21], sheep [34], goat [30] and buffalo [29] (Fig. 3, Additional file 1: Table S4). In contrast, *ARSF*, a member of the ARS family, has been reported as a PAR gene in other mammalian species, but is not found in any of the ruminant PARs [29, 30, 34].

### Comparison of X-degenerate Y chromosome regions

**Fig. 4:**
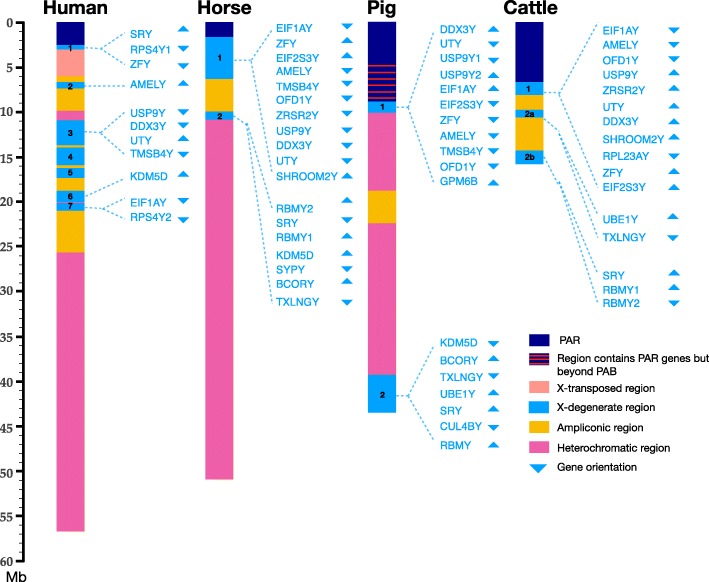
Comparison of Y chromosome X-degenerate regions in cattle and other mammals. The human Y chromosome is different from other mammals with 7 X-d regions and one unique X-transposed region (pink). Eight X-d1 genes are shared among cattle, pig and horse but the gene order and orientation are not the same. The cattle X-d2 is split into and X-d2a X-d2b regions with 2 and 3 genes respectively. The cattle Y chromosome diagram does not include the unknown heterochromatic regions

Most of the X-Y paired genes of cattle, pig and horse that are outside the PAR are found in the X degenerate region, X-d1, located adjacent to the PAR (Fig. 4). Of the 11 genes in the cattle X-d1 region 8 are in common with horse and pig X-d1 regions, but the gene order differs between the three species. *RPL23AY* is only found in the cow X-d1, while *TMSB4Y* is found in the horse and pig X -d1 regions and the human X-d3 region which is missing from cow X-d regions. Five additional bovine gametologs are found in two X-d2 regions, X-d2a and X-d2b, which correspond to the single X-d2 in horse and pig. Cattle X-d2a is distal to X-d1 and contains 2 genes, *UBE1Y* and *TXLNGY*. Both genes are found in the pig X-d2 region but *UBE1Y* is in an ampliconic region of the horse Y chromosome. The cattle X-d2b region contains SRY and is in a telomeric position similar to the X-d2 region of pig. The cattle X-d2b region contains two copies of *RBMY*, which is also duplicated in the horse X-d2 [20].

## Discussion

The trio-binning approach facilitated the construction of the most complete bovine X and Y chromosome sequence assemblies available to date. Alignment of the Brahman cattle X with Hereford X revealed a major inverted region. In both assemblies this inverted region ends at contig breakpoints and could be an assembly artefact rather than a true biological difference. We could not resolve this discrepancy using RH and linkage maps [35–37] because the marker density was insufficient. Alignments of the Brahman cattle X chromosome with those of the water buffalo, goat and sheep, revealed a high level of co-linearity, but with numerous break points and several inversions. Many of these structural differences are consistent with gross karyotypic rearrangements that differentiate goat and sheep X chromosomes from those of cattle and water buffalo [38]. The evolutionary separation of goat and sheep from cattle and buffalo occurred 20–25 MYBP, which pre-dates the separation of goat and sheep from each other, 10–15 MYBP, and of buffalo from cattle, about 12 MYBP [39]. This evolutionary history is consistent with the differences in X chromosome structure we see among these species [24, 40].

The X chromosomes of human, pig, dog and horse have a strikingly similar pattern of differences relative to the Brahman cattle X chromosome. Ruminants are even-toed ungulates which separated from odd-toed ungulates (including the pig and horse) more than 60 MYBP, suggesting that the rearrangements occurred after this split but prior to the separation of the ruminants, about 25 MYBP [24].

Comparison of the Brahman cattle X chromosome with other mammalian reference genomes suggest the X chromosome assemblies for the Hereford cattle, sheep, goat and pig are incomplete at the proximal end of the PAR. The proximal PAR genes of Brahman cattle, water buffalo, human and horse are the same. Genes missing from the proximal end of the Hereford cattle X chromosome have been misplaced on various autosomes, whereas the genes missing from sheep, goat and pig, are found on unplaced contigs. Given that the common ancestor of cattle, water buffalo, human and horse existed about 96 MYBP, it is more parsimonious to suggest the gene order and structure of the proximal PAR are conserved and not assembly artefacts. The assembly of the Brahman cattle and water buffalo [29] X chromosomes may have benefited from the use of much longer PacBio reads and improved assembly algorithms.

The distal PAR boundary in ancient species is thought to lie within the *AMEL* locus [41]. However, there was no substantial identity of the Brahman X and Angus Y chromosomes for the region between 2933 bp distal to *GPR143* and *AMELX*. The PAR boundary in cattle is therefore just distal to *GPR143*. The distal ends of the PARs of water buffalo and sheep also lie close to *GRP143.*

The expansion of *OBP* and *BDA20* gene families in ruminants suggests they have a specific role in these species. These genes are members of the lipocalin family which are involved in immune-modulation and anti-inflammatory responses [42]. For example, their expression changes after exposure of cattle to ticks [43]. The ARS gene family in the ruminant PAR is missing *ARSF* that is found in non-ruminant species, suggesting that it has been lost during ruminant evolution.

Recombination of mammalian sex chromosomes only occurs in the PAR. This may explain why the MSY regions are rich in repetitive sequences [2, 44]. The co-linearity between Angus and Hereford Y chromosomes is limited to X-d and *PRAMEY* regions, while the numerous repetitive sequences in ampliconic regions appear expanded in the Hereford Y. This may be due to the use of various BAC clones from several individuals to assemble the Hereford Y chromosome [45]. The Angus Y chromosome assembly shows some alignment in some isolated areas with pig, horse and human Y chromosomes, which is mainly in X-degenerate and ampliconic regions. This is consistent with rapid evolution of non-recombining Y chromosome sequences [3].

We identified 16 X-Y paired genes in 3 X-degenerate regions on the Angus Y chromosome, which were interspersed by ampliconic regions that contain multiple copies of bovine-specific *PRAMEY, ZNF280AY* and *ZNF280BY* genes. *PRAMEY* genes are exclusively expressed in testis and are involved in spermatogenesis during testicular maturation [46]. ZNF280BY and ZNF280AY are multi-copy Y-genes transposed from an autosome. The temporal and spatial expression patterns of these genes also suggests that they play a role in spermatogenesis [47]. While horse, pig and cattle X-d1 are similar in gene content, gene order is very different in the three species. *RPL23AY* is cattle-specific and a second copy of this gene, *RPL23A*, is found on chromosome 19, located in a conserved block in mammals with intron-exon structure and six identical intron-less pseudogenes with 91% sequence identity to this gene are found on cattle chromosomes 3, 9, 10, 14, 22 and 29, suggesting that these copies have arisen by retrotransposition.

Cattle have lost several ancestral Y genes from X-d regions including *KD5MD*, *TMSB4Y* and *TXLNGY*. Ten of the 16 gametologs are conserved in the X-d regions of horse, 8 in pig and 5 in human. The genes in the X-d regions, do not recombine, and hence diverge over time, allowing for the possibility of sex-specific selection. They may therefore be involved in sexual dimorphism [48]. Five of these (*DDX3Y*, *EIF1AY*, *USP9Y*, *UTY*, *ZFY*) are global regulators of gene activity expressed across a broad range of adult tissues and could have profound effects on sexual development [48].

## Conclusions

The quality of assemblies achieved for the X chromosome, and for the PAR, X-d and ampliconic regions of the Y chromosome, using the trio-binning approach, enabled us to examine and compare major structures of the bovine sex chromosomes, both between the sex chromosomes and among species. Alignment of the Brahman X and Angus Y chromosomes precisely identified boundaries and gene content of the PAR region and indicated that the proximal end of the PAR is truncated in the sheep, goat and current bovine reference genome assemblies. The sequence data revealed expansions of gene families in the ruminant PAR region that have previously been associated with immune function, and conservation of gametologs that are known dosage sensitive regulators of gene expression. The sex chromosome assemblies and the annotation presented are valuable resources for the molecular characterization of sex-specific phenotype in livestock and other species.

## Methods

### Sample collection

All animal work was approved by the University of Adelaide Animal Ethics Committee (No. S-094-2005). Briefly, a Brahman cow was bought by the University of Adelaide from a farm (Kiowa, Kingstown, New South Wales) and was transported to SARDI experimental farm at Struan South Australia where it was inseminated with semen of an Angus bull bought by the University from American Breeder Services, Australia. At day 153 post-insemination, the cow was humanely killed by stunning and exsanguination at a commercial abattoir (Dalriada Abattoir, Keith, South Australia) as per standard operating procedures. The uterus was recovered and the male fetus removed and immediately humanely killed by stunning and exsanguination. Lung tissue was collected and snap frozen in liquid nitrogen. Details of the contig creation for this assembly using the trio binning method have been previously described [23]. Briefly, DNA was extracted from fetal lung, paternal semen, and maternal uterine tissue. Long-read libraries of the fetus were prepared for sequencing on the Sequel platform as suggested by the manufacturer (Pacific Biosciences, Menlo Park, CA). Short-read libraries of the sire and dam were prepared for sequencing on the NextSeq500 platform as recommended by the manufacturer (Illumina, Inc. San Diego, CA). Approximately 60x short-read coverage of the dam and sire were produced, and 134x long-read coverage for the fetus. Parent-specific kmers were identified, long reads sorted into bins by parental origin, and independent haploid assemblies constructed using triocanu (implemented in Canu v1.7).

### Sex chromosomes assembly and validation

Haplotype resolved paternal and maternal contigs constructed using Canu [23] were scaffolded independently using Hi-C reads [49] and an optical map (Bionano tools v1.3.0), which were then consolidated into chromosome specific groups of scaffolds and the sex chromosome scaffolds selected for the male and female assembly. The high density of repetitive elements on both the X and Y chromosomes made assembly difficult, breaking sequence contiguity even with long sequence reads, so additional markers were used to validate the order and orientation of scaffolds. For the X chromosome, the USDA-MARC Bovine linkage Map [35], and two RH maps, the BovGen RH map [37] and SUNbRH7000-rad map [36] were used to place, order and orientate scaffolds. For the Y chromosome, known genes in cattle [16, 22], pig (Sscrofa11.1) and human (GRCh38.p12) assemblies were used to identify Y-specific scaffolds. Cattle Y chromosome RH map markers [50] were used to guide ordering and orientation of the Y-specific scaffolds. To exclude scaffolds incorrectly identified as Y sequence, 26 scaffolds which were shorter than 50 kb and contained fewer than three known Y genes, were manually inspected. These shorter scaffolds were partitioned into 50-kb bins and aligned with the CHORI-240 Bovine BAC library Y specific clones (see URLs) using BLASTN. Six sequences with less 90% alignment were removed. Further details on X and Y chromosomes scaffolds identification and orientation is given in Additional file 1: Note 1 and 2, respectively.

### Conflict resolution

The RH X chromosome marker order and orientation was generally in agreement with the X chromosome assembly. However, 15 out of 84 markers from the BovGen RH map and 18 out of 93 markers from the SUNbRH7000-rad map suggested a possible assembly error. Comparison of the RH maps showed that 5 markers from BovGen RH map and 4 markers from SUNbRH7000-rad map had a consistent arrangement that differed from the Brahman X assembly. Both RH maps showed an inversion of a 1,286,607 bp contig in a scaffold that is made up of 4 contigs. With the exception of this scaffold, other RH marker inconsistencies would have required contigs to be broken and rearranged. The Brahman X and ARS-UCD1.2 X agreed in all these regions so the order and orientation of scaffolds that agreed best with Hi-C and optical map was retained.

Outside of the PAR, the Y chromosome assembly is made up of small scaffolds with average length of 98,509 bp. The ordering of scaffolds was completely dependent on the 52 RH markers and no contig breaks were required.

### X and Y chromosome alignments

In order to compare intra- and inter-species sex chromosomes differences, pairwise alignments were carried out for cattle (Brahman, Angus, Hereford, Nelore), water buffalo, goat, sheep, human, pig, dog and horse using the aligner Lastz v1.04 [51] with default settings. Brahman X is reverse complement to Hereford X. Prior to alignments, the Hereford X and Nelore X are reverse orientated for comparison. Repeats in the X and Y chromosomes were masked by Repeatmasker v4–0-7 using cow RepBase23.08 [52]. The number of gaps was calculated using a custom python script. The number of inversions were calculated by Smash with a 1 Mb block size [53]. Further details on the alignment parameters is given in Additional file 1: Note 3.

### X and Y chromosome gene annotations

All X chromosome genes from ARS-UCD1.2 were remapped to the Brahman X chromosome using the Exonerate v2.4 software [54]. For the PAR, detailed manual annotation was carried out. A total of 42 protein-coding PAR genes were used to annotate the X chromosome. These genes came from the following sources: 1) 15 human PAR1 genes [8]; 2) 22 candidate cattle PAR genes [11]; 3) horse [55], dog [56] and pig [57] PAR genes. A cattle PAR gene, arylsulfatase E (*ARSE*), was reported in a FISH mapping study [11] but the sequence is not available from NCBI. However, three other arylsulfatases paralogs on the X chromosome were available and were used to search for other similar members on the PAR.

The current cattle reference genome assembly ARS-UCD1.2 does not have a Y chromosome, therefore 18 Y-specific genes from the X-degenerate regions were selected from a study of Y chromosome transcribed sequences [16], in addition, MSY genes from pig and human Y chromosomes were used for the Angus Y chromosome annotation. Further details on gene annotation are given in Additional file 1: Note 4 and Additional file 1: Table S1.

### Conservation of synteny in mammalian PARs

Each of the X and Y chromosomes PARs were contained in single scaffold. To estimate the boundary of PAR, X and Y scaffolds containing PARs were partitioned into 1 Mb windows and aligned against each other using BLASTN. From the alignments, the first bin from the proximal end that had coverage below 80% and percentage identity below 80% was identified as the pseudoautosomal boundary. The gene order of PAR genes between closely related species was analysed using Bioconductor Gviz plots [58].

## Supplementary information

**Additional file 1: Figure S1.** Alignment of the Brahman X with other mammalian X chromosomes. **Figure S2.** Alignment of the Angus cattle Y against other mammalian Y chromosomes. **Figure S3.** Multi-copy genes in Angus Y ampliconic region **Table S1.** Summary of X and Y chromosome protein-coding genes. **Table S2.** Copy numbers of the protein-coding genes in the bovine MSY. **Table S3.** List of PAR genes missing in the current Hereford, sheep, goat and pig assemblies. **Table S4.** Copy numbers of OBP and BDA20 genes in each species.

## Data Availability

Sequence data is available from the BioProject Accession PRJNA432857. Analysis of Angus and Brahman genome assemblies, https://github.com/lloydlow/BrahmanAngusAssemblyScripts; the CHORI-240 Bovine BAC Library is available from https://bacpacresources.org/filtersAvail.php,; ArrowGrid, https://github.com/skoren/ArrowGrid; RepeatMasker, http://www.repeatmasker.org.

## References

[1] Ohno S (1969). Evolution of sex chromosomes in mammals. Annu Rev Genet.

[2] Bull JJ (1983). Evolution of sex determining mechanisms: the Benjamin/Cummings publishing company, Inc.

[3] Graves JA (2006). Sex chromosome specialization and degeneration in mammals. Cell.

[4] Charlesworth B (1991). The evolution of sex chromosomes. Science.

[5] Graves JA (1998). Evolution of the mammalian Y chromosome and sex-determining genes. J Exp Zool.

[6] Skaletsky H, Kuroda-Kawaguchi T, Minx PJ, Cordum HS, Hillier L, Brown LG, Repping S, Pyntikova T, Ali J, Bieri T (2003). The male-specific region of the human Y chromosome is a mosaic of discrete sequence classes. Nature.

[7] NaG E-M (2008). J.A.M: X and Y Chromosomes: Homologous Regions.

[8] Helena Mangs A, Morris BJ (2007). The human Pseudoautosomal region (PAR): origin, Function and Future. Curr Genomics.

[9] Hughes JF, Skaletsky H, Pyntikova T, Minx PJ, Graves T, Rozen S, Wilson RK, Page DC (2005). Conservation of Y-linked genes during human evolution revealed by comparative sequencing in chimpanzee. Nature.

[10] Van Laere AS, Coppieters W, Georges M (2008). Characterization of the bovine pseudoautosomal boundary: documenting the evolutionary history of mammalian sex chromosomes. Genome Res.

[11] Das PJ, Chowdhary BP, Raudsepp T (2009). Characterization of the bovine pseudoautosomal region and comparison with sheep, goat, and other mammalian pseudoautosomal regions. Cytogenet Genome Res.

[12] Johnson T, Keehan M, Harland C, Lopdell T, Spelman RJ, Davis SR, Rosen BD, Smith TPL, Couldrey C (2019). Short communication: identification of the pseudoautosomal region in the Hereford bovine reference genome assembly ARS-UCD1.2. J Dairy Sci.

[13] Gabriel-Robez O, Rumpler Y, Ratomponirina C, Petit C, Levilliers J, Croquette MF, Couturier J (1990). Deletion of the pseudoautosomal region and lack of sex-chromosome pairing at pachytene in two infertile men carrying an X;Y translocation. Cytogenet Cell Genet.

[14] Mohandas TK, Speed RM, Passage MB, Yen PH, Chandley AC, Shapiro LJ (1992). Role of the pseudoautosomal region in sex-chromosome pairing during male meiosis: meiotic studies in a man with a deletion of distal Xp. Am J Hum Genet.

[15] Hughes JF, Skaletsky H, Pyntikova T, Graves TA, van Daalen SK, Minx PJ, Fulton RS, McGrath SD, Locke DP, Friedman C (2010). Chimpanzee and human Y chromosomes are remarkably divergent in structure and gene content. Nature.

[16] Chang TC, Yang Y, Retzel EF, Liu WS (2013). Male-specific region of the bovine Y chromosome is gene rich with a high transcriptomic activity in testis development. Proc Natl Acad Sci U S A.

[17] Hughes JF, Skaletsky H, Brown LG, Pyntikova T, Graves T, Fulton RS, Dugan S, Ding Y, Buhay CJ, Kremitzki C (2012). Strict evolutionary conservation followed rapid gene loss on human and rhesus Y chromosomes. Nature.

[18] Soh YQ, Alfoldi J, Pyntikova T, Brown LG, Graves T, Minx PJ, Fulton RS, Kremitzki C, Koutseva N, Mueller JL (2014). Sequencing the mouse Y chromosome reveals convergent gene acquisition and amplification on both sex chromosomes. Cell.

[19] Skinner BM, Sargent CA, Churcher C, Hunt T, Herrero J, Loveland JE, Dunn M, Louzada S, Fu B, Chow W (2016). The pig X and Y chromosomes: structure, sequence, and evolution. Genome Res.

[20] Janecka JE, Davis BW, Ghosh S, Paria N, Das PJ, Orlando L, Schubert M, Nielsen MK, Stout TAE, Brashear W (2018). Horse Y chromosome assembly displays unique evolutionary features and putative stallion fertility genes. Nat Commun.

[21] Liu Y, Qin X, Song XZ, Jiang H, Shen Y, Durbin KJ, Lien S, Kent MP, Sodeland M, Ren Y (2009). Bos taurus genome assembly. BMC Genomics.

[22] Canavez FC, Luche DD, Stothard P, Leite KR, Sousa-Canavez JM, Plastow G, Meidanis J, Souza MA, Feijao P, Moore SS (2012). Genome sequence and assembly of Bos indicus. J Hered.

[23] Koren Sergey, Rhie Arang, Walenz Brian P, Dilthey Alexander T, Bickhart Derek M, Kingan Sarah B, Hiendleder Stefan, Williams John L, Smith Timothy P L, Phillippy Adam M (2018). De novo assembly of haplotype-resolved genomes with trio binning. Nature Biotechnology.

[24] Hiendleder S, Lewalski H, Janke A (2008). Complete mitochondrial genomes of Bos taurus and Bos indicus provide new insights into intra-species variation, taxonomy and domestication. Cytogenet Genome Res.

[25] Consortium BG, Elsik CG, Tellam RL, Worley KC, Gibbs RA, Muzny DM, Weinstock GM, Adelson DL, Eichler EE, Elnitski L (2009). The genome sequence of taurine cattle: a window to ruminant biology and evolution. Science.

[26] Loftus RT, MacHugh DE, Bradley DG, Sharp PM, Cunningham P (1994). Evidence for two independent domestications of cattle. Proc Natl Acad Sci U S A.

[27] LWPdL FA (2007). Mapping of the bovine Y chromosome. Electron J Biol.

[28] Kuderna LFK, Lizano E, Julia E, Gomez-Garrido J, Serres-Armero A, Kuhlwilm M, Alandes RA, Alvarez-Estape M, Juan D, Simon H (2019). Selective single molecule sequencing and assembly of a human Y chromosome of African origin. Nat Commun.

[29] Low WY, Tearle R, Bickhart DM, Rosen BD, Kingan SB, Swale T, Thibaud-Nissen F, Murphy TD, Young R, Lefevre L (2019). Chromosome-level assembly of the water buffalo genome surpasses human and goat genomes in sequence contiguity. Nat Commun.

[30] Bickhart DM, Rosen BD, Koren S, Sayre BL, Hastie AR, Chan S, Lee J, Lam ET, Liachko I, Sullivan ST (2017). Single-molecule sequencing and chromatin conformation capture enable de novo reference assembly of the domestic goat genome. Nat Genet.

[31] Qiu Q, Zhang G, Ma T, Qian W, Wang J, Ye Z, Cao C, Hu Q, Kim J, Larkin DM (2012). The yak genome and adaptation to life at high altitude. Nat Genet.

[32] Bana NA, Nyiri A, Nagy J, Frank K, Nagy T, Steger V, Schiller M, Lakatos P, Sugar L, Horn P (2018). The red deer Cervus elaphus genome CerEla1.0: sequencing, annotating, genes, and chromosomes. Mol Gen Genomics.

[33] Ge RL, Cai Q, Shen YY, San A, Ma L, Zhang Y, Yi X, Chen Y, Yang L, Huang Y (2013). Draft genome sequence of the Tibetan antelope. Nat Commun.

[34] Archibald AL, Cockett NE, Dalrymple BP, Faraut T, Kijas JW, Maddox JF, JC ME, Hutton Oddy V, Raadsma HW, International Sheep Genomics C (2010). The sheep genome reference sequence: a work in progress. Anim Genet.

[35] Ihara N, Takasuga A, Mizoshita K, Takeda H, Sugimoto M, Mizoguchi Y, Hirano T, Itoh T, Watanabe T, Reed KM (2004). A comprehensive genetic map of the cattle genome based on 3802 microsatellites. Genome Res.

[36] Itoh T, Watanabe T, Ihara N, Mariani P, Beattie CW, Sugimoto Y, Takasuga A (2005). A comprehensive radiation hybrid map of the bovine genome comprising 5593 loci. Genomics.

[37] Jann OC, Aerts J, Jones M, Hastings N, Law A, McKay S, Marques E, Prasad A, Yu J, Moore SS (2006). A second generation radiation hybrid map to aid the assembly of the bovine genome sequence. BMC Genomics.

[38] Iannuzzi L, King WA, Di Berardino D (2009). Chromosome evolution in domestic bovids as revealed by chromosome banding and FISH-mapping techniques. Cytogenet Genome Res.

[39] Kumar S, Stecher G, Suleski M, Hedges SB (2017). TimeTree: a resource for timelines, Timetrees, and divergence times. Mol Biol Evol.

[40] Arnason U, Janke A (2002). Mitogenomic analyses of eutherian relationships. Cytogenet Genome Res.

[41] Iwase M, Satta Y, Hirai Y, Hirai H, Imai H, Takahata N (2003). The amelogenin loci span an ancient pseudoautosomal boundary in diverse mammalian species. Proc Natl Acad Sci U S A.

[42] Logdberg L, Wester L (2000). Immunocalins: a lipocalin subfamily that modulates immune and inflammatory responses. Biochim Biophys Acta.

[43] Wang YH, Reverter A, Kemp D, McWilliam SM, Ingham A, Davis CA, Moore RJ, Lehnert SA (2007). Gene expression profiling of Hereford shorthorn cattle following challenge with Boophilus microplus tick larvae. Aust J Exp Agric.

[44] Rice WR (1996). Evolution of the Y sex chromosome in animals. Bioscience.

[45] Rozen S, Warren WC, Weinstock G, OBrein S (2006). J GRWRKPD: sequencing and annotating new mammalian Y chromosomes a white paper proposal.

[46] Liu WS, Zhao Y, Lu C, Ning G, Ma Y, Diaz F, O'Connor M (2017). A novel testis-specific protein, PRAMEY, is involved in spermatogenesis in cattle. Reproduction.

[47] Yang Y, Chang TC, Yasue H, Bharti AK, Retzel EF, Liu WS (2011). ZNF280BY and ZNF280AY: autosome derived Y-chromosome gene families in Bovidae. BMC Genomics.

[48] Bellott DW, Hughes JF, Skaletsky H, Brown LG, Pyntikova T, Cho TJ, Koutseva N, Zaghlul S, Graves T, Rock S (2014). Mammalian Y chromosomes retain widely expressed dosage-sensitive regulators. Nature.

[49] Ghurye J, Rhie A, Walenz BP, Schmitt A, Selvaraj S, Pop M, Phillippy AM, Koren S. Integrating Hi-C links with assembly graphs for chromosome-scaleassembly. bioRxiv 2019. p. 261149. 10.1371/journal.pcbi.1007273 PMC6719893 31433799

[50] Liu WS, Mariani P, Beattie CW, Alexander LJ, Ponce De Leon FA (2002). A radiation hybrid map for the bovine Y chromosome. Mamm Genome.

[51] Harris RS. Improved pairwise alignment of genomic DNA. Ph.D. Thesis, The Pennsylvania State University; 2007.

[52] Bao W, Kojima KK, Kohany O (2015). Repbase update, a database of repetitive elements in eukaryotic genomes. Mob DNA.

[53] Pratas D, Silva RM, Pinho AJ, Ferreira PJ (2015). An alignment-free method to find and visualise rearrangements between pairs of DNA sequences. Sci Rep.

[54] Slater GS, Birney E (2005). Automated generation of heuristics for biological sequence comparison. BMC Bioinformatics.

[55] Moore SS, Byrne K, Johnson SE, Kata S, Womack JE (2001). Physical mapping of CSF2RA, ANT3 and STS on the pseudoautosomal region of bovine chromosome X. Anim Genet.

[56] Young AC, Kirkness EF, Breen M (2008). Tackling the characterization of canine chromosomal breakpoints with an integrated in-situ/in-silico approach: the canine PAR and PAB. Chromosom Res.

[57] Skinner BM, Lachani K, Sargent CA, Affara NA (2013). Regions of XY homology in the pig X chromosome and the boundary of the pseudoautosomal region. BMC Genet.

[58] Hahne F, Ivanek R, Mathé E, Davis S (2016). Visualizing Genomic Data Using Gviz and Bioconductor. Statistical Genomics: Methods and Protocols.

