# Supplementary material for: New insights into mammalian sex chromosome structure and evolution using high-quality sequences from bovine X and Y chromosomes
Source: BMC Genomics. 2019 Dec 19;20:1000. doi: 10.1186/s12864-019-6364-z (PMC6923926; doi:10.1186/s12864-019-6364-z)
Supplement: Supplementary file 1 — Additional file 1: Figure S1. Alignment of the Brahman X with other mammalian X chromosomes. Figure S2. Alignment of the Angus cattle Y against other mammalian Y chromosomes. Figure S3. Multi-copy genes in Angus Y ampliconic region Table S1. Summary of X and Y chromosome protein-coding genes. Table S2. Copy numbers of the protein-coding genes in the bovine MSY. Table S3. List of PAR genes missing in the current Hereford, sheep, goat and pig assemblies. Table S4. Copy numbers of OBP and BDA20 genes in each species. [file 12864_2019_6364_MOESM1_ESM.docx]

**Additional file**

| **Supplementary Notes** | | 1 |
| --- | --- | --- |
| 1) X chromosome scaffolds identification and orientation | | 1 |
| 2) Y chromosome scaffolds identification and orientation | | 2 |
| 3) Comparison of X and Y chromosomes in mammals | | 2 |
| 4) Gene annotation of sex chromosomes | | 3 |
|  |  |  |
| **Supplementary Figures** | | 4 |
| 1. Alignments of Brahman X against other X chromosomes   a) Brahman X vs Cattle (Hereford: ARS-UCD1.2) X  b) Brahman X vs Water Buffalo (UOA_WB_1) X  c) Brahman X vs Goat (ARS1) X  d) Brahman X vs Sheep (Oar_v4.0) X  e) Brahman X vs Pig (Sscrofa11.1) X  f) Brahman X vs Dog (CanFam3.1) X  g) Brahman X vs Horse (EquCab3.0) X  h) Brahman X vs Human (GRCH38.p12) X | | 4 |
| 1. Alignments of Angus Y against other Y chromosomes   a) Angus Y vs Human (eMSYv3.1) Y  b) Angus Y vs Horse (MH341179) Y  c) Angus Y vs Pig (Sscrofa11.1) Y | | 6 |
| 3) Multi-copy genes in Angus Y ampliconic region | | 7 |
| **Supplementary Tables** | | 8 |
| 1) Summary of X and Y chromosome protein-coding genes  2) Copy numbers of the protein-coding genes in the bovine MSY | | 8  9 |
| 3) Missing PAR genes from four species listed in figure 3 | | 10 |
| 4) Copy numbers of OBP and BDA20 genes in each species | | 12 |

**Supplementary Notes**

1. X chromosome scaffolds identification and orientation

X chromosome RH and linkage map markers[1-3] were aligned to the maternal (Brahman) contigs using blastn[4] with the following parameters*:* (*-max_hsps 3 -perc_identity 90 -qcov_hsp_perc 80*). Contigs were ordered based on the linkage and RH map marker order and then used to assess the concordance with scaffolds based on Hi-C and optical maps. Three Hi-C scaffolds were broken and re-joined based on the RH map order. There was no conflict between optical map and RH map. The Brahman X chromosome was scaffolded by optical mapping (17 scaffolds consisting of 107 contigs) and by Hi-C (14 scaffolds consisting of 57 contigs). The additional optical mapping contigs had a total length of 10 Mb. Hi-C scaffolds were longer, on average, than optical map based scaffolds. As the optical map based scaffolds were in closer agreement with the linkage and RH data, their scaffolds were joined together based on Hi-C scaffolds. This was then polished, gap filled[5] and the consensus sequence rebuilt with ArrowGrid (<https://github.com/skoren/ArrowGrid>). The final length of the X chromosome was 146,092,946 Mb.

1. Y chromosome scaffolds identification and orientation

The Y chromosome contigs were initially identified by the presence of genes reported on the Y chromosomes on the bovine genome assemblies (Bos indicus 1.0, Btau 5.0.1) and a previous cattle Y chromosome gene expression study[6]. We then aligned Y chromosome SNP probes from bovine HD 50k chip and 62 Y-linked RH map markers[7, 8] to paternal contigs and confirmed that 91 contigs originated from cattle Y chromosome. Six contigs with small number of Y chromosome SNP probes were not included as Y sequence because they were in conflict with optical map scaffolding, which placed them on large 4 autosomal scaffolds. In rest of 73 contigs, six contigs were removed as they have <80% sequencing identify in alignment with CHORI-240 Bovine BAC library Y. The Hi-C and optical map scaffolds were ordered and orientated based on RH map markers to produce scaffolds that were in best agreement. The final length of the Y chromosome was 15,658,480 Mb. This was then subjected to gap filling[5] and consensus sequence rebuilding with ArrowGrid.

1. Comparison of X and Y chromosomes in mammals

To facilitate comparison of sex chromosomes across mammalian species representative reference genomes were downloaded from NCBI, these were X (cattle, water buffalo, sheep, human, pig, dog horse) and Y chromosome (cattle, human, pig) were. For the goat X chromosome, we manually joined two X chromosome unplaced scaffolds in the goat genome (NW_017189516.1 and NW_017189517.1) and aligned them to our Brahman X. Horse Y chromosome sequences were obtained from Horse eMSYv3.1 assembly (GenBank MH341179)[9]. Prior to alignments, repeats in the Brahman X and Angus Y chromosomes were masked by Repeatmasker v4-0-7 using cow RepBase23.08[10]. Repeat-masked sex chromosomes from other species were downloaded from the NCBI. Pairwise alignments were generated using the aligner Lastz v1.04[11] with the following parameters.

For intra species*: --notransition --step=20 --nogapped --format=maf --ambiguous=iupac\*

For inter species: *--notransition --step=50 --nogapped --format=maf --ambiguous=iupac\*

1. Gene annotation of sex chromosomes

To annotate the X chromosome, we downloaded mRNA sequences from the cattle assemblies in NCBI (ARS-UCD 1.2 and Bos indicus 1.0) and lifted these over to the Brahman X using Exonerate v2.4.0[12] with a cut off of 88% (parameters: *--model est2genome --querytype dna--targettype dna--showvulgar no --showalignment no --showtargetgff yes --showcigar no --percent 88*). A total of 983 genes were annotated on the Brahman X. To annotate the Y chromosome, we downloaded the mRNA from cattle Y chromosome (Btau 5.0.1), previous cattle Y chromosome sequence[6] and other mammalian orthologous sequences (human, pig and horse) and aligned these against the Angus Y assembly. We lifted cattle Y chromosome sequences over using same parameters as we used for Brahman X. In addition to cattle Y genes, other homologous Y genes from human, pig, and horse were used as input to search for Y genes in Angus using 75% sequence identity as cut off. In total, 51 unique genes were annotated in Angus Y including genes on PAR.

**Figure S1**: Alignment of the Brahman X with other mammalian X chromosomes.

The Brahman X chromosome is displayed on the x-axes and the X chromosomes from other species on the y-axes. The panels show alignments with a) Cattle (Hereford) X from ARS-UCD1.2 assembly. Two major inversion blocks (>1 Mb) are highlighted in yellow. b) Water Buffalo X from UOA_WB_1 assembly. c) Goat X from ARS1 assembly. d) Sheep X from Oar_v4.0 assembly. e) Pig X from Sscrofa11.1 assembly. f) Dog X from CanFarm3.1 assembly. g) Horse X from EquCab3.0 assembly. h) Human X from GRCH38.p12 assembly.

**Figure S2**: Alignment of the Angus cattle Y against other mammalian Y chromosomes

The Angus Y chromosome is on the x-axes and the Y chromosomes from other species on the y-axes. Panels are a) Human Y from GRCH38.p12 assembly. b) Horse Y from eMSYv3.1 assembly. c) Pig Y from Sscrofa11.1 assembly.

**Figure S3**: Multi-copy genes in Angus Y ampliconic region

The ampliconic region are from 8.07Mb to 10.01Mb and 10.81Mb to 14.05Mb. The triangular dot plot showing the location of gene copies in Angus Y ampliconic region.

**
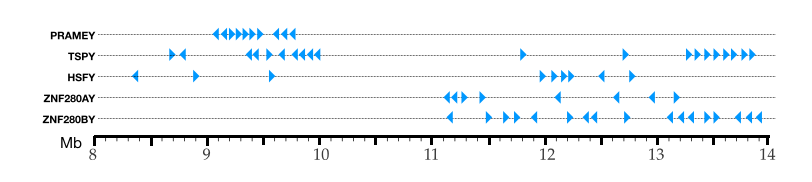
**

**Table S1**: Summary of X and Y chromosome protein-coding genes

| X Genes | Total | Single copy | Multi-copy |
| --- | --- | --- | --- |
| X-specific | 936 | 936 | |
| PAR | 31 | 29 | 2 |
| X-Y paired outside PAR | 16 | 16 | - |
| Total genes | 983 | 981 | 2 |
| Total transcripts | 988 | 981 | 7 |
| Y Genes | Total | Single copy | Multi-copy |
| Y-specific | 4 | 0 | 4 |
| PAR | 31 | 29 | 2 |
| X-Y paired outside PAR | 15 | 14 | 1 |
| Total genes | 51 | 43 | 6 |
| Total copies | 153 | 43 | 113 |

A total of 983 genes were annotated on the X chromosome by lift over from three cattle assemblies (ARS-UCD1.2, Bos indicus 1.0 and Btau 5.0.1). These included 936 X-specific single/multiple copy genes, 31 PAR genes and 16 X-Y paired genes. Lift over of Y chromosome annotated genes from previous cattle Y chromosome sequences[6] identified a total of 51 genes including 31 PAR genes, 15 X-Y paired genes and four Y-specific genes without X-homologues. Total number genes on Y including PAR genes are 153. These genes were used as input for Exonerate v2.4.0 to search the Brahman X and Angus Y chromosomes.

**Table S2**: Copy numbers of the protein-coding genes in the bovine MSY

| Y Genes (X-d region) | copies |
| --- | --- |
| EIF1AY | 1 |
| AMELY | 1 |
| OFD1Y | 1 |
| USP9Y | 1 |
| ZRSR2Y | 1 |
| UTY | 1 |
| DDX3Y | 1 |
| SHROOM2Y | 1 |
| RPL23AY | 1 |
| ZFY | 1 |
| EIF2S3Y | 1 |
| UBE1Y | 1 |
| TXLNGY | 1 |
| SRY | 1 |
| RBMY | 2 |
| Y Genes (ampliconic region) | Total |
| PRAMEY | 10 |
| HSFY | 9 |
| TSPY | 20 |
| ZNF280AY | 8 |
| ZNF280BY | 17 |

This is the summary of copy number of known MSY genes. there are 15 genes in X-d regions and 5 known multi-copy genes in ampliconic region.

**Table S3**: List of PAR genes missing in the current Hereford, sheep, goat and pig assemblies

| PAR genes | Hereford | | Sheep | | Goat | | Pig | |
| --- | --- | --- | --- | --- | --- | --- | --- | --- |
|  | Chr | Accession version | Chr | Accession version | Chr | Accession version | Chr | Accession version |
| PLCXD1 | Chr1 | NM_001105044.1 | ChrUn | XM_004022871.2 | ChrUn | XM_018044435.1 | - | - |
| GTPBP6 | Chr1 | XM_024995411.1 | ChrUn | XM_012107966.1 | ChrUn | XM_018044432.1 | - | - |
| PPP2R3B | Chr1 | XM_024995403.1 | ChrUn | XM_012107967.1 | ChrUn | XM_018044433.1 | ChrUn | XM_021081364.1 |
| SHOX | Chr2 | NM_001191546.2 | - | - | ChrUn | XM_018045110.1 | ChrUn | XM_021081396.1 |
| CRLF2 | Chr29 | XM_024987475.1 | ✓ | ✓ | ChrUn | XM_005701362.3 | ChrUn | XM_021081383.1 |
| CSF2RA | Chr3 | XM_024990295.1 | ✓ | ✓ | ChrUn | XM_005701352.3 | ChrUn | XM_021081383.1 |
| IL3RA | Chr3 | XM_024990300.1 | ✓ | ✓ | ChrUn | XM_018044557.1 | ChrUn | XR_002341032.1 |
| SLC25A6 | Chr26 | NM_174660.2 | ✓ | ✓ | ChrUn | XM_018044555.1 | ChrUn | NM_214418.2 |
| ASMTL | Chr26 | NM_001035058.1 | ✓ | ✓ | ChrUn | XM_018044443.1 | ChrUn | XM_021081390.1 |
| P2RY8 | Chr3 | XM_005228542.3 | ✓ | ✓ | ChrUn | XM_013976840.2 | ChrUn | XM_021081386.1 |
| AKAP17A | Chr3 | XM_024990316.1 | ✓ | ✓ | ChrUn | XM_018044444.1 | ChrUn | XM_021081363.1 |
| ASMT | Chr1 | NM_177493.2 | ✓ | ✓ | ChrUn | NM_001285598.1 | ChrUn | XM_021081386.1 |
| DHRSX | ✓ | ✓ | ✓ | ✓ | ChrUn | XM_018044442.1 | ChrY | XM_021080887.1 |
| ZBED1 | ✓ | ✓ | ✓ | ✓ | ChrUn | XM_018044439.1 | ChrUn | XM_021082454.1 |
| CD99 | ✓ | ✓ | ✓ | ✓ | ✓ | ✓ | ChrUn | XR_002340897.1 |
| XG | ✓ | ✓ | ✓ | ✓ | ✓ | ✓ | ✓ | ✓ |
| GYG2 | ✓ | ✓ | ✓ | ✓ | ✓ | ✓ | ✓ | ✓ |
| ARSD | ✓ | ✓ | ✓ | ✓ | ✓ | ✓ | ✓ | ✓ |
| ARSE | ✓ | ✓ | ✓ | ✓ | ✓ | ✓ | ✓ | ✓ |
| ARSH | ✓ | ✓ | ✓ | ✓ | ✓ | ✓ | - | - |

The PAR genes missing from the cattle (Hereford) assembly were found on five autosomes (chromosome 1,2,3,26, 29). Missing PAR genes in the in sheep, goat and pig assemblies were found either in unplaced scaffolds or as follows: sheep SHOX and pig

GTPBP6, PPP2R3B and ARSH were not found in their current X chromosome assembly. IL3RA and CD99 are among the non-coding RNA in the current pig assembly.

**Table S4**: Copy numbers of OBP and BDA20 genes in each species

|  | Brahman | Hereford | Water buffalo | Sheep | Goat | Pig | Dog | Horse |
| --- | --- | --- | --- | --- | --- | --- | --- | --- |
| OBP | ENSBIXG00005001147 | XM_024988434.1 | XM_025276408.1 | XM_027963189.1 | XM_018043719.1 | NM_213796.1 | XM_025440685.1 | XM_014728749.1 |
|  | \|  \| ENSBIXG00005011676 \| \| --- \| --- \| | XM_005228543.4 | XM_025276466.1 | \|  \| XM_027963188.1 \| \| --- \| --- \| | XM_018044049.1 | XM_021080597.1 | \|  \| XM_005640968.2 \| \| --- \| --- \| | XM_014728750.1 |
|  | ENSBIXG00005001130 | XM_002700469.6 | XM_025276502.1 | - | - | - | XM_025435144.1 | - |
|  | - | XM_024988435.1 | XM_025276828.1 | - | - | - | - | - |
|  | - | \|  \| XM_010800829.3 \| \| --- \| --- \| | - | - | - | - | - | - |
| BDA20 | ENSBIXG00005011448 | XM_024988433.1 | \|  \| XM_006074786.2 \| \| --- \| --- \| | XM_012106177.2 | XM_018043718.1 | - | - | - |
|  | ENSBIXG00005011472 | XM_010822282.3 | XM_025276762.1 | XM_006074786.2 | XM_018044027.1 | - | - | - |
|  | ENSBIXG00005011425 | \|  \| XM_010800830.3 \| \| --- \| --- \| | XM_025276829.1 | - | XM_005701239.2 | - | - | - |
|  | ENSBIXG00005011448 | - | - | - | XM_018044047.1 | - | - | - |

There are varies copy number of OBP and BDA20 among species. OBP found in all species we compared in this paper except human. BDA20 only found in ruminant species. 2 copies in sheep, three copies in Goat and 4 copies in both brahman and Hereford.

**Supplementary References**

1. Jann OC, Aerts J, Jones M, Hastings N, Law A, McKay S, Marques E, Prasad A, Yu J, Moore SS *et al*: **A second generation radiation hybrid map to aid the assembly of the bovine genome sequence**. *BMC Genomics* 2006, **7**:283.

2. Ihara N, Takasuga A, Mizoshita K, Takeda H, Sugimoto M, Mizoguchi Y, Hirano T, Itoh T, Watanabe T, Reed KM *et al*: **A comprehensive genetic map of the cattle genome based on 3802 microsatellites**. *Genome Res* 2004, **14**(10A):1987-1998.

3. Itoh T, Watanabe T, Ihara N, Mariani P, Beattie CW, Sugimoto Y, Takasuga A: **A comprehensive radiation hybrid map of the bovine genome comprising 5593 loci**. *Genomics* 2005, **85**(4):413-424.

4. Camacho C, Coulouris G, Avagyan V, Ma N, Papadopoulos J, Bealer K, Madden TL: **BLAST+: architecture and applications**. *BMC Bioinformatics* 2009, **10**:421.

5. English AC, Richards S, Han Y, Wang M, Vee V, Qu J, Qin X, Muzny DM, Reid JG, Worley KC *et al*: **Mind the gap: upgrading genomes with Pacific Biosciences RS long-read sequencing technology**. *PLoS One* 2012, **7**(11):e47768.

6. Chang TC, Yang Y, Retzel EF, Liu WS: **Male-specific region of the bovine Y chromosome is gene rich with a high transcriptomic activity in testis development**. *Proc Natl Acad Sci U S A* 2013, **110**(30):12373-12378.

7. Liu WS, Mariani P, Beattie CW, Alexander LJ, Ponce De Leon FA: **A radiation hybrid map for the bovine Y Chromosome**. *Mamm Genome* 2002, **13**(6):320-326.

8. Stafuzza NB, Abbassi H, Grant JR, Rodrigues-Filho EA, Ianella P, Kadri SM, Amarante MV, Stohard P, Womack JE, de Leon FA *et al*: **Comparative RH maps of the river buffalo and bovine Y chromosomes**. *Cytogenet Genome Res* 2009, **126**(1-2):132-138.

9. Janecka JE, Davis BW, Ghosh S, Paria N, Das PJ, Orlando L, Schubert M, Nielsen MK, Stout TAE, Brashear W *et al*: **Horse Y chromosome assembly displays unique evolutionary features and putative stallion fertility genes**. *Nat Commun* 2018, **9**(1):2945.

10. Bao W, Kojima KK, Kohany O: **Repbase Update, a database of repetitive elements in eukaryotic genomes**. *Mob DNA* 2015, **6**:11.

11. Harris RS: **Improved pairwise alignment of genomic DNA.** *PhD Thesis, The Pennsylvania State University* 2007.

12. Slater GS, Birney E: **Automated generation of heuristics for biological sequence comparison**. *BMC Bioinformatics* 2005, **6**:31.
